# Perioperative complications and short-term outcomes of abdominal sacrocolpopexy, laparoscopic sacrocolpopexy, and laparoscopic pectopexy for apical prolapse

**DOI:** 10.1590/S1677-5538.IBJU.2017.0692

**Published:** 2018

**Authors:** Alper Biler, I. Egemen Ertas, Gokhan Tosun, Ismet Hortu, Unal Turkay, Ozge E. Gultekin, Gulfem Igci

**Affiliations:** 1 Department of Obstetrics and Gynecology, University of Health Sciences Tepecik Training and Research Hospital, Izmir, Turkey; 2 Department of Obstetrics & Gynecology, Ege University School of Medicine, Izmir, Turkey; 3 Department of Obstetrics and Gynecology, University of Health Sciences Kocaeli Derince Training and Research Hospital, Kocaeli, Turkey; 4 Department of Statistics, Ege University, Izmir, Turkey

**Keywords:** Laparoscopy, Pelvic Organ Prolapse, complications [Subheading]

## Abstract

**Objective:**

To investigate differences in perioperative complications and short-term outcomes of patients who underwent abdominal sacrocolpopexy / sacrohysteropexy, laparoscopic sacrocolpopexy / sacrohysteropexy, or laparoscopic pectopexy due to apical prolapse.

**Materials and Methods:**

A retrospective cohort study was performed on 110 patients who underwent apical prolapse surgery between January 1, 2011, and July 31, 2017. Only symptomatic uterine or vaginal vault prolapse patients with stage 2-4, according to the pelvic organ prolapse quantification system, were included. Baseline and intraoperative variables of groups; perioperative complications, including hemorrhage, urinary, and wound complications, blood transfusion, ileus, and short-term outcomes were compared.

**Results:**

A total of 68 abdominal sacrocolpopexies (44 sacrocolpopexies and 24 sacrohysteropexies), 14 laparoscopic sacrocolpopexies (10 sacrocolpopexies and 4 sacrohysteropexies), and 28 laparoscopic pectopexies (16 pectopexies and 12 pectohysteropexies) were analyzed. Baseline characteristics and intraoperative variables were similar. However, the mean operating time was significantly shorter in the laparoscopic pectopexy group (74.9 min) when compared with that of the other groups (p < 0.01). During the six-month follow-up period, no prolapse recurrence and mesh erosion / exposure were observed in any group. *De-novo* stress urinary incontinence, urgency, and defecation problems, as well as perioperative complication rates, were not statistically significantly different between the groups.

**Conclusions:**

Although the complication rates and short-term outcomes were not significantly different between the groups, minimally invasive approaches were associated with reduced procedural-related morbidity. Laparoscopic pectopexy is a promising endoscopic prolapse surgery and can be an alternative technique to sacrocolpopexy.

## INTRODUCTION

Pelvic organ prolapse (POP) affects millions of women worldwide and is a health problem for 50% of parous women over 50 years old (1). POP surgery is one of the most common procedures of benign gynecological surgery. Many different surgical procedures have been described over time for correcting this situation (2). Although there is no consensus as to the finest surgical approach, the most accepted options for apical compartment prolapse, meaning the downward displacement of the vaginal apex, uterus, or cervix, are vaginal sacrospinous ligament fixation and abdominal sacrocolpopexy / sacrohysteropexy.

Abdominal sacrocolpopexy is a well-known technique in POP treatment and is considered the gold standard for surgical treatment of apical compartment prolapse (3). According to the Cochrane collaboration review, abdominal sacrocolpopexy is associated with high long-term success rates compared to vaginal sacrospinous ligament fixation (3). However, compared with vaginal surgery, abdominal sacrocolpopexy is associated with longer operating times, longer recovery times and higher costs (3). In addition, the higher morbidity associated with abdominal sacrocolpopexy led to a search for less invasive abdominal approaches. Along with these investigations, improvements in minimally invasive surgery led to the introduction of the laparoscopic and, subsequently, the robot-assisted laparoscopic approaches to abdominal sacrocolpopexy (4). Today, laparoscopic sacrocolpopexy, with the advantages of reduced morbidity and a short duration of hospital stay, offers outcomes as good as abdominal sacrocolpopexy in the correction of apical compartment prolapse (5, 6).

Recently, a new laparoscopic technique for prolapse surgery specifically developed for obese patients, called pectopexy, was presented by Bannerjee and Noe (7). In this technique, the lateral parts of the iliopectineal ligament are used for bilateral mesh fixation of the descended structures, and the mesh follows round and broad ligaments without crossing the ureter or bowel (7). In the light of the latest literature; a laparoscopic pectopexy has been used as an alternative method in patients having difficult and complicated promontorium dissection during surgery (7-10).

The goal of the present study was to investigate the differences in perioperative complications and short-term outcomes of patients who underwent abdominal sacrocolpopexy / sacrohysteropexy, laparoscopic sacrocolpopexy / sacrohysteropexy, and laparoscopic pectopexy due to symptomatic apical prolapse.

## MATERIALS AND METHODS

Medical records were retrospectively reviewed on all women who underwent surgery for apical prolapse performed by the same surgeons at two teaching hospitals between January 1, 2011, and July 31, 2017. The present cohort study was conducted in accordance with the ethical standards of the Declaration of Helsinki and received institutional review board approval.

The extent of the uterine or vaginal vault prolapse was determined by a gynecological examination and ultrasound, and we used the POP quantification system (POP-Q) for prolapse assessment. In order to assess the influence of pressure, the patients were examined in both a lying and sitting position; this assessment was important to avoid an overcorrection or undercorrection. Only symptomatic uterine or vaginal vault prolapse patients with POP-Q stage 2 and above were included. Symptoms include a sensation of pressure on the vagina and perineum, seeing and feeling a bulge / protrusion in the distal vagina, chronic lower back pain, dyspareunia and other sexually related problems, or associated lower urinary tract symptoms including urgency, frequency, urinary retention, and incontinence. Patients having a contraindication for surgery, previous operations for vaginal prolapse correction, pelvic inflammatory disease, or previously identified or strongly suspected massive adhesions between the sigmoid colon and the presacral peritoneal area were excluded from the study. Demographic data, including age at surgery, parity, menopausal status, body mass index (BMI), and comorbidities, as well as perioperative information such as the type of surgical procedure, total operating time, estimated blood loss, and duration of hospital stay, were obtained from the patient's electronic medical records. A preoperative evaluation for occult stress urinary incontinence (SUI) was performed through clinical cough urinary stress testing with and without reduction of prolapse to all continent women with apical prolapse. Urodynamic studies were performed in women with complicated SUI.

The major factor for deciding the type of surgery was increasing surgical experience over the years in terms of laparoscopic surgery. The other factors were BMI and comorbidities of the patients. We performed laparoscopic pectopexy on patients who had a BMI greater than 30. Moreover, pectopexy was chosen for patients who experienced difficulty during laparoscopic promontorium dissection due to a fatty presacral area, the presence of unexpected vessel variations, or a less flexible and thick sigmoid colon. In addition, patients having comorbidities like chronic obstructive / restrictive lung disease and who were not suitable for general anesthesia underwent abdominal sacrocolpopexy with regional anesthesia by the recommendation of the anesthesiology team.

A perioperative complication was described as any complication that happened during surgery or within 6 to 10 weeks postoperatively, including injury to the bladder, bowel, vagina, ureters, or vessels; wound complications; hematoma; abscess; urinary tract infection; bowel obstruction; ileus; blood transfusion; and mesh infection. Short-term outcome was used to describe the six-month period after surgery. Estimated blood loss was calculated by measuring the difference between pre- and postoperative hemoglobin levels. The operating time calculated excluded time elapsed for simultaneous procedures such as an anterior colporrhaphy, posterior colporrhaphy, and culdoplasty, but did include time elapsed for a hysterectomy. The duration of hospital stay was measured from admission to discharge. The at-risk patients received antithrombotic prophylaxis with low molecular-weight heparin. Antibiotic prophylaxis was administrated in all patients. Women having vaginal erosion were prescribed a vaginal estrogen cream two to three weeks before surgery. The urethral catheter was removed on the first postoperative day. All patients returned for follow-up examinations at least two times during the six-month period after the surgery and evaluated for subjective and anatomical outcome. To document defecation disorders, the defecation section of the International Consultation on Incontinence Questionnaire was used.

## OPERATIVE PROCEDURES

### Abdominal or laparoscopic sacrocolpopexy / sacrohysteropexy

The key steps of the abdominal and laparoscopic sacrocolpopexy / sacrohysteropexy procedures were the same. If an SUI or multiple pelvic floor defects were present, both open and laparoscopic sacrocolpopexy were typically combined with other surgical procedures such as anterior / posterior colporrhaphy, culdoplasty, or Burch colposuspension. In patients who had not already undergone a hysterectomy, a total hysterectomy was performed first.

For abdominal sacrocolpopexy, the peritoneal cavity was entered with a Pfannenstiel incision or a low midline laparotomy incision. After entering the peritoneal cavity, the hysterectomy was performed if necessary. If the uterus had been previously removed, after distinguishing the vaginal vault, its covering peritoneum was dissected. To attach the mesh, a sufficiently broad area was exposed in the superior aspects of the pubocervical and rectovaginal fascia. Then, the peritoneal layer over the promontory was incised vertically. The loose areolar tissues were gently dissected to the posterior cul-de-sac, avoiding damage to the rectum and the ureter. A type 1 monofilament polypropylene mesh was attached to the anterior and posterior vagina distally and to the anterior longitudinal ligament proximally at the level of the promontory using non-absorbable sutures. The mesh was then reperitonealized. In patients with the uterus preserved, a transverse incision at the posterior surface of the uterus, where the sacrouterine ligaments are attached, was performed for a sacrohysteropexy.

All laparoscopic operations were performed using standard endoscopic equipment. A 10 mm trocar was inserted from the umbilicus for the scope, and three additional 5 mm ports were inserted. One of these was placed 5-6 cm left of the umbilicus, and the other two were placed 2 cm medial and superior to the anterior superior iliac crests. A RUMI© uterine manipulator (Cooper Surgical, Trumbull, CT, US) was introduced vaginally at the beginning of the procedure, which was the same as the abdominal procedure.

### Laparoscopic pectopexy

The standard laparoscopic preparation was done as above. After a 10 mm trocar was inserted from the umblicus for the scope, three additional 5 mm ports were inserted under direct visualization of the lower intra-abdominal area, median, left, and right, from 2 cm medial and superior to the anterior superior iliac crests. In patients who were to undergo hysterectomy, a total hysterectomy was performed first. As in sacrocolpopexy, if multiple pelvic floor defects were present, we frequently combined this procedure with other surgical procedures (e.g., anterior / posterior colporrhaphy, paravaginal repair).

We performed the pectopexy procedure as previously described by Banerjee and Noe (7 First, we opened the peritoneal layer along the right round ligament toward the pelvic side wall. An incision was made with the harmonic scalpel in the medial and caudal direction, and the right external iliac vein was visualized. Soft tissue in this area was dissected with blunt dissection so that an approximately 4-5 cm segment of the right iliopectineal ligament (Cooper's ligament) adjacent to the insertion of the iliopsoas muscle could be identified. The same procedure was then repeated on the left side of the patient. The peritoneal layers on both sides were opened toward the vaginal apex, and the anterior and posterior areas of the vaginal apex were prepared for the mesh fixation. In patients with a preserved uterus, the anterior peritoneum of the uterus was dissected, and the lower anterior segment of the uterus was prepared for the mesh fixation. After completion of dissections, a type 1 monofilament polypropylene mesh was inserted into the abdominal cavity. The ends of the mesh were sutured to both iliopectineal ligaments using the intracorporeal suture technique with nonabsorbable sutures. The mesh, in the tension-free position, was fixed to the vaginal apex or uterus with polydioxanone sutures, and the vaginal apex or uterus was provided with a hammock-like fixation. Finally, the peritoneum above the mesh was sutured with an absorbable suture material.

### Statistical analysis

In the present study, descriptive statistics were obtained for the variables in the three groups. The comparisons between the groups for quantitative variables (age, BMI, parity, operating time, preop Hb, postop Hb, and hospital stay) were tested using the Kruskal-Wallis test, which is a nonparametric test. For the qualitative variables (menopause status, comorbidities, prior surgery, prolapsus, complications), a chi-square analysis (which is also a nonparametric test) was used to determine whether there was a significant difference between the three groups. When the assumption of normality is not met and sample sizes are not large enough, nonparametric tests are an alternative to parametric tests. For this study, the significance level was 0.01. SPSS 20 (Statistical Package for the Social Sciences, IBM, Armonk, NY, US) was used for data analysis.

## RESULTS

A total of 110 patients with apical prolapse who fulfilled the inclusion criteria were evaluated and subsequently surgically treated. Sixty-eight patients underwent abdominal sacrocolpopexy (sacrocolpopexy (n = 44); sacrohysteropexy (n = 24)), 14 patients underwent laparoscopic sacrocolpopexy (sacrocolpopexy (n = 10); sacrohysteropexy (n = 4)), and 28 patients underwent laparoscopic pectopexy (pectopexy (n = 16); pectohysteropexy (n = 12)). Of the 110 patients, 40 patients (36.3%) had vaginal vault prolapse, 24 in the abdominal sacrocolpopexy group, 4 in the laparoscopic sacrocolpopexy group, and 12 in the pectopexy group. There were 33 patients with stage 2 POP, 48 patients with stage 3 POP, and 29 patients with stage 4 POP. Table-1 shows the patient's characteristics. No significant differences were observed between the groups.

**Table 1:** Patients’ preoperative characteristics in the study groups.

		Abdominal sacrocolpopexy (n=68)	Laparoscopic sacrocolpopexy (n=14)	Laparoscopic pectopexy (n=28)	P
Age (years) *		52.8 ± 12.1	53.7 ± 11.8	48.3 ± 12.7	0.194
Parity*		3.5 ± 1.6	2.9 ± 1.1	2.8 ± 1.1	0.192
BMI (kg/m^2^) *		26.9 ± 1.9	25.9 ± 1.9	26.1 ± 3.1	0.022
Menopause**					0.339
	Yes	42 (61.8)	9 (64.3)	13 (46.4)	
	No	26 (38.2)	5 (35.7)	15 (53.6)	
Comorbidities**					0.171
	Yes	23 (33.8)	8 (57.1)	8 (28.6)	
	No	45 (66.2)	6 (42.9)	20 (71.4)	
History of prior pelvic surgery**					0.268
	Yes	25 (36.8)	7 (50)	15 (53.6)	
	No	43 (63.2)	7 (50)	13 (46.4)	
History of prior prolapsus surgery**			1 (7.1)	4 (14.3)	0.756
	Yes	7 (10.3)	13 (92.9)	24 (85.7)	
	No	61 (89.7)			

Values are expressed as * = mean ± SD and ** n (%). BMI, body mass index.

Table-2 shows perioperative data for the study groups. The mean operating time was 74.96 min in the laparoscopic pectopexy group versus 117.35 min in the abdominal sacrocolpopexy group and 178.57 min in the laparoscopic sacrocolpopexy group (p < 0.01). The hospital stay, preoperative and postoperative hemoglobin levels, and surgical procedures performed simultaneously were not significantly different between the groups. Concomitant anti-incontinence surgery was performed in nine patients with stress incontinence. A concomitant retropubic incontinence procedure (Burch colposuspension) was preferred in patients with abdominal and laparoscopic sacrocolpopexy, whereas a mid-urethral sling procedure (transobturator tape) was performed in patients with laparoscopic pectopexy.

**Table 2:** Perioperative characteristics and performed surgical procedures in the study groups.

		Abdominal sacrocolpopexy (n=68)	Laparoscopic sacrocolpopexy (n=14)	Laparoscopic pectopexy (n=28)	P
Operating time (min.)*		117.3 ± 41.6	178.5 ± 50.5	74.9 ± 34.05	**<0.01**
Preoperative Hb (g/dL)*		12.8 ± 1.1	12.1 ± 0.9	12.8 ± 1.3	0.049
Postoperative Hb (g/dL)*		11.04 ± 1.05	10.6 ± 0.9	11.4 ± 1.1	0.04
Hospital stay (days)*		2.3 ± 0.92	2 ± 0.5	2 ± 0.01	0.048
**Surgical procedures**					
	Sacrocolpopexy**	44	10	–	
	Sacrohysteropexy**	24	4	–	
	Pectopexy**	–	–	16	
	Pectohysteropexy**	–	–	12	
	Abdominal hysterectomy**	20	–	–	
	Laparoscopic hysterectomy**	–	6	3	
	Anterior colporrhaphy**	5	1	2	
	Posterior colporraphy**	11	3	3	
	Culdoplasty**	65	11	–	
	Burch colposuspension**	8	1	–	
	Trans obturator tape**	–	–	1	
	Cervical coll amputation**	4	–	–	
	Vaginal paravaginal repair**	–	–	3	

Values are expressed as * = mean ± SD and ** = n.

Complications were seen in nine patients (13.2%) in the abdominal sacrocolpopexy group, one patient (7.1%) in the laparoscopic sacrocolpopexy group, and one patient (3.6%) in the pectopexy group. However, the complication rates of each group did not differ significantly (p = 0.332). There was no operative mortality in any group. The complications are listed in Table-3. Wound infection was the most common complication in the patients who underwent abdominal sacrocolpopexy. Conversion to laparotomy was not required for any patient in the laparoscopic sacrocolpopexy group or the laparoscopic pectopexy group. Hemorrhage from presacral veins occurred in two patients, one of which occurred during laparoscopic sacrocolpopexy and the other during abdominal sacrocolpopexy. Use of a Z-suture and application of pressure with a warm sponge were sufficient to stop the bleeding. These patients did not require a blood transfusion. In the abdominal sacrocolpopexy group, one patient had a mild ileus, but no additional treatment was required in this case.

**Table 3:** perioperative complications in the study groups.

		Abdominal sacrocolpopexy (n=68)	Laparoscopic sacrocolpopexy (n=14)	Laparoscopic pectopexy (n=28)	P
Overall complications					0.332
	Yes	9 (13.2)	1 (7.1)	1 (3.6)	
	No	59 (86,8)	13 (92.9)	27 (96.4)	
Hemorrhage		1 (1.4)	1 (7.1)	1 (1.7)	
Urinary infection		2 (2.9)	–	–	
Febrile morbidity		1 (1.4)	–	–	
Wound infection		3 (4.4)	–	–	
Wound dehiscence		1 (1.4)	–	–	
Mild ileus		1(1.4)	–	–	
Intraoperative injury		–	–	–	

The subjective satisfaction rates were high in all patients at one week and at six months postoperatively. At the six-month follow-up, there had been no occurrences of de novo apical prolapse, anterior or lateral defect cystoceles, rectoceles, or mesh erosion / exposure in any group. However, one dyspareunia, four de novo urgency, and two de novo SUI cases occurred in the abdominal sacrocolpopexy group. One de novo persistant constipation, one de novo urgency, and one de novo SUI case occurred in the laparoscopic sacrocolpopexy group. Two de novo urgency and one de novo SUI case occurred in the laparoscopic pectopexy group. Data demonstrating the short-term outcome was presented in Table-4.

**Table 4:** Short-term follow-up outcome in the study groups.

	Abdominal sacrocolpopexy (n=68)	Laparoscopic sacrocolpopexy (n=14)	Laparoscopic pectopexy (n=28)	P
Subjective satisfation	67 (98.5)	14 (100)	27 (96.4)	0.162
Apical prolapse recurrence	0	0	0	–
*De novo* urgency	4 (5.8)	1 (7.1)	2 (7.1)	0.456
*De novo* SUI	2 (2.9)	1 (7.1)	1 (3.5)	0.361
*De novo* persistant constipation	0	1 (7.1)	0	0.194
*De novo* dyspareunia	1 (1.4)	0	0	0.292

All values are expresed as n (%). SUI = Stress urinary Incontinence

## DISCUSSION

We conducted a retrospective cohort study with 110 patients who underwent abdominal sacrocolpopexy, laparoscopic sacrocolpopexy, and laparoscopic pectopexy for apical prolapse. The current study demonstrates that the pectopexy group had shorter operation times. The complication rates and short-term outcomes were not significantly different between there groups. However, a tendency was seen toward fewer complications in the laparoscopic sacrocolpopexy and pectopexy groups.

Sacrocolpopexy has been performed laparoscopically for over 20 years (6, 11). However, the procedure is still associated with some problems. Although it depends on the surgeon's skill, the difficult surgical field at the ventral side of the sacrum is one of the stressed issues. Therefore, many surgeons have modified the technique and have fixed the mesh to the top of the promontory (9, 12), which results in a positional change in direction to the abdominal wall at the vaginal axis. Another important point is that when working in the area of the sacrum, care should be taken to avoid damage to the sigmoid, presacral veins, and right ureter (10).

In 2007, Banerjee and Noe described a new method of endoscopic prolapse surgery, laparoscopic pectopexy, that was specifically developed for obese patients due to the difficulty of presacral area dissection (7). Laparoscopic pectopexy is not associated with a high intraoperative risk because, with this technique, the mesh follows round and broad ligaments without crossing the ureter or bowel, and because there is no narrowing of the pelvic outlet, defecation disorders are not expected. In addition, the hypogastric vessels and nerves are also a safe distance from any danger (7). Noe et al. compared short-term operative outcomes of laparoscopic pectopexy and sacrocolpopexy procedures in a randomized comparative study of 83 patients with symptomatic primary vaginal prolapse POP-Q ≥ 2 (8). They showed that the mean operating time and blood loss were significantly lower in the pectopexy group. No major complications occurred in either group, and there was no significant difference in the hospital stay. The authors emphasized that laparoscopic pectopexy is a good alternative to laparoscopic sacrocolpopexy in cases of an especially difficult surgical field. More recently, Kale et al. shared their first experience with laparoscopic pectopexy (10), successfully performing the surgery in symptomatic apical prolapse patients with POP-Q stage 2 and above without intraoperative or postoperative complications. Over the past three years, we have also been performing the pectopexy technique described by Banerjee and Noé (7, 8) for apical prolapse surgery. In our practice, we have been choosing pectopexy progressively for apical prolapse surgery with the rising surgical experience. Similar to previous studies, in our study, the mean operating times were shortest in the pectopexy group (p < 0.01).

The most worrying intraoperative complication of sacrocolpopexy is hemorrhage from the presacral vessels, which may have life-threatening consequences (13). Bladder injuries, blood transfusions, and wound complications are other complications related to the procedure. Nygaard et al. published a comprehensive review of 3.827 cases of abdominal sacrocolpopexy (14). Among these cases, the incidence of bladder injuries was 3.1%; hemorrhages, blood transfusions, or both was 4.4%; wound complications, including infections, hematomas, or superficial separation was 4.6%; and urinary infection was 10.9%. In our study, although there is a tendency for wound complications in abdominal group, overall complication rates were not significantly different between there groups.

Defecation problems and de novo SUI ranging from 17-37% and 4-50%, respectively, are the most frequently reported complications associated with sacrocolpopexy (15, 16). The placement of the mesh between the sacrum and vagina (cervix) always narrows the pelvis, and the cause of defecation problems may be the reduced space in the pelvis or trauma to the hypogastric nerves (12, 14, 15). In our cohort, de novo constipation was not observed in the pectopexy group, which is in accordance with previously published data (7-10). However, de novo urgency and de novo SUI rates were comparable in both groups. We found low de novo SUI rates of 2.9% (abdominal sacropexy), 7.1% (laparoscopic sacropexy), and 3.5% (pectopexy). This may be explained by our site-specific multicompartment surgical strategy and avoiding too much traction. Moreover, the recurrence rates for apical prolpasus were not statistically significant different between there groups and these data are in concordance with previous published data (7-10).

Laparoscopic procedures were associated with longer operating times, longer learning curves, and higher costs than either abdominal or vaginal surgery (3, 17, 18). Paraiso et al. analyzed 117 patients with vaginal cuff prolapse and concluded that the mean operating time was significantly greater in the laparoscopy group than in the abdominal sacrocolpopexy group (17). Furthermore, the hospital stay was significantly decreased in the laparoscopy group. After excluding the time elapsed for simultaneous procedures such as anti-incontinence and vaginal surgeries for each group, the mean operating times were significantly longer in the laparoscopic sacrocolpopexy group, similar to previously published data. Although the hospital stay was longer in the abdominal sacrocolpopexy group, the length of stay was not significantly different between the groups.

To our knowledge, this is the first study to compare perioperative results of various surgical methods, including abdominal-laparoscopic sacrocolpopexy and laparoscopic pectopexy, used in apical prolapse surgery. Studies evaluating the perioperative complications and outcomes of these procedures are not commonly performed, and only a few reports have been published (8, 19). In addition, most studies emphasized long-term outcomes and the efficacy of the procedures (5, 9, 12, 14, 17). In the present study, there were more complications in the abdominal sacrocolpopexy group, but these were not significantly different between the groups (p = 0.332). Most of the complications in this group were wound complications. Similar to the literature, the mean operating times were shortest in the pectopexy group.

The main limitation of the present study is its retrospective nature and the small sample size, especially in the laparoscopic sacrocolpopexy group. Retrospective cohort studies are subject to selection bias, recall bias, and unknown confounding variables, which may negatively affect the accuracy of the results. Another limitation is the relatively short-term follow-up of patients postoperatively. We tracked outcomes for six months postoperatively, which may underestimate the incidence of recurrence of POP, de novo SUI, or mesh erosion. The strengths of the present study are the inclusion and evaluation of three different surgical routes and having no patients lost to follow-up in six months.

## CONCLUSIONS

Abdominal sacrocolpopexy, laparoscopic sacrocolpopexy, and laparoscopic pectopexy have comparable perioperative complications and short-term anatomical and subjective outcomes. Although the complication rates were not significantly different between the groups, the laparoscopic sacrocolpopexy and pectopexy groups had less morbidity. Moreover, laparoscopic pectopexy is a novel promising method for POP correction that offers some practical advantages, such as shorter operating times when compared with laparoscopic sacrocolpopexy, so that it can be added to a surgeon's methods to more adequately react in complex presacral area dissections. Finally, present study can burden the knowledge and shed some light on future prospective studies with larger sample sizes demonstrating the long-term outcomes of laparoscopic pectopexy.
